# The roles, responsibilities and perceptions of community health workers and ward-based primary health care outreach teams: a scoping review

**DOI:** 10.1080/16549716.2020.1806526

**Published:** 2020-09-07

**Authors:** Euphemia Mbali Mhlongo, Elizabeth Lutge, Lateef Adepeju

**Affiliations:** a School of Nursing & Public Health, University of KwaZulu- Natal, Durban, South Africa; b KwaZulu-Natal Department of Health, Pietermaritzburg, South Africa

**Keywords:** Scoping review, community health workers, ward-based primary health care outreach teams, perceptions and roles

## Abstract

**Background:**

Community health workers play important roles in rural primary health care settings. They work within ward-based primary health care outreach teams yet their roles are not always clearly defined and operationalized. There is thus a need to explore perceptions about the roles and responsibilities of community health workers.

**Objective:**

To investigate the roles, responsibilities and effectiveness of community health workers working within ward-based primary health care outreach teams.

**Method:**

A scoping review of the published peer reviewed literature on community health workers working in ward-based primary health care outreach teams within low and middle-income countries was conducted. The following five electronic databases were searched: EBSCOhost, Google Scholar, Science Direct, EMBASE, PubMed, and Clinical key. Out of 69,969 identified articles, 31 met the inclusion criteria. The majority of the studies were from South Africa.

**Results:**

Both positive and negative perceptions were reported. Suggestions for improvements were also put forward. Positive factors included: ongoing training and up skilling; collaboration and trust building with other health care workers; mentoring and supervision; motivation and recognition, and incentives and remuneration. Negative factors covered: inadequate mentorship and poor supervision; role conflict; lack of support; poor remuneration; inadequate manpower; poor knowledge, and insufficient training. The review identified the following as the roles and responsibilities of community health workers: conducting home visits; identifying vulnerable community groups; promoting health and wellness; increasing access to health care; contact tracing; delivering health education; giving counselling and psychosocial support, and providing preventive health services.

**Conclusion:**

The information available for community health workers in terms of their roles, responsibilities and effectiveness is inadequate, considering their roles and responsibilities in ward-based primary health care outreach teams. This lack of information constitutes barriers to effective service delivery, a common situation among this group of community health workers.

## Background

Globally the jobs of community health workers (CHWs) cover a broad range of activities such as linking individuals to health care services, increasing awareness about health through outreach advocacy, undertaking home visits, acting as intermediaries between the community and health professionals and helping to manage patients in their communities [1]. The extent of these workers’ education and professional training varies across settings. Assisting with the provision of health care to communities has become a more significant aspect of their work in recent decades [2]. It is important to understand the roles and responsibilities of CHWs in order to ensure that they function effectively within their teams [3].

The prevalence of communicable and non-communicable diseases such as respiratory diseases, diabetes mellitus, HIV/AIDS, cancers and cardiovascular diseases requires early diagnosis to prevent complications and death [4]. CHWs have been recognised for the important role they play in strengthening primary health care [4,5] especially in low- and middle-income countries (LMICs). There is a need for greater understanding of their roles and responsibilities. This knowledge can help enhance community participation and improve the quality of the services delivered by CHWs [6].

In the 1940s a team headed by Sidney Kark and his wife established a programme involving the delivery of community-oriented primary care in the region of Pholela, KwaZulu-Natal, South Africa. This community-oriented primary health programme (COPC) has continued to develop since that time and currently supports the primary health care system in South Africa and the way in which CHWs work [7,8]. Ideally ward-based primary health care outreach teams in South Africa and other LMICs comprise six CHWs, an outreach team leader who is a registered nurse, a promotion officer and an environmental health officer [9].

CHWs deliver extensive services and have broad ranging responsibilities. These include visiting homes and reporting health-related information with regard to the individual, family and community [10]. In the Brazilian health care system CHWs form the foundation of the health care system, and this model is currently being adopted in South Africa. The importance of CHWs as members of community-based health programmes therefore cannot be overstated, and there is a need for greater understanding of their roles and responsibilities.

## Aim of the study

To investigate the roles, responsibilities and effectiveness of community health workers working within ward-based primary health care outreach teams.

## Methods

A comprehensive account of the scoping review methodology followed in this study has already been published [11] in response to the research question: What are the roles and responsibilities of ward-based primary health care outreach teams in KwaZulu-Natal?

This scoping review adopted the Arksey and O’Malley framework and its five steps as its governing process [12]. Below are the steps:

1. Identifying the research question.
2. Identifying relevant studies.
3. Study selection.
4. Charting the data.
5. Collating, summarising and reporting the results.

In addition, the report of the review followed the Preferred Reporting Items for System Review Guideline (PRISMA) [13].

## Search Strategy

An extensive search for eligible articles was conducted on the following databases: Science Direct, Google Scholar, EMBASE, Clinical key and EBSCOhost platform. On EBSCOhost the search included: PsycINFO, PubMed, Medline, Health Source: Nursing Academic Edition, CINAHL, Educational Source, and Health Source. Essentially, the search strategy focused on the population, concept and context (PCC), as guided by The Joanna Briggs Institute Reviewers’ Manual, 2015 [14]. See Table 1 below. The Boolean operators (AND, OR) were also used to separate the keyword/s during the search. The supplementary file contains the summary of the search strategy.

**Table 1.** PCC framework.

Criteria	Determinants
Population	Community health worker
Concept	Ward-based primary health care outreach teams OR primary health care OR community home-based care OR community health care OR health care delivery in rural settings.
Context	Low- and middle- income countries, e.g. South Africa.

## Eligibility criteria

### Inclusion criteria

Studies reporting on WBPHCOTs.

Studies reporting on CHWs.

Studies published in the English language.

### Exclusion criteria

Studies not conducted in LMICs.

Study protocols.

Letters to editors and opinion pieces.

Studies reporting on review articles.

## Study selection

**Figure 1.**
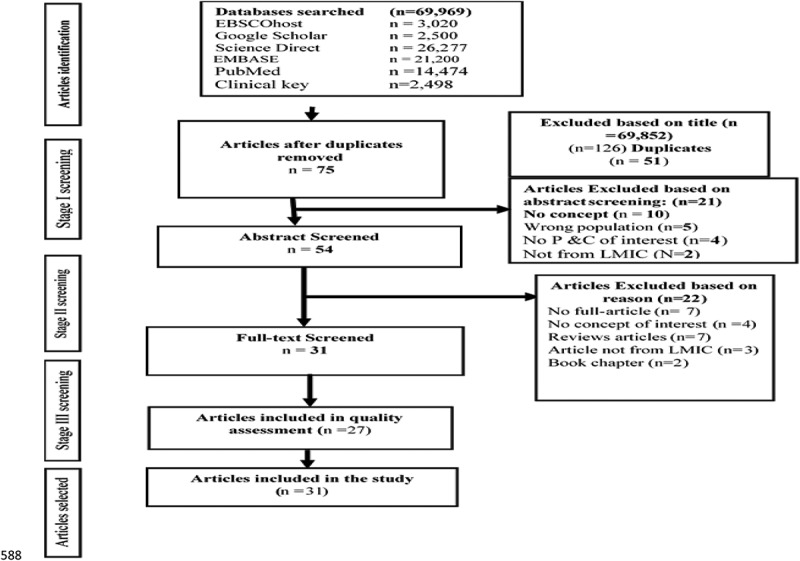
Flow chart of the search strategy results adapted from PRISMA (2009).

The process for the selection of the included articles involved three phases of screening that were rigorously conducted before data extraction: the title screening phase, the abstract phase and the full-text screening phase. The title screening was conducted by EM alone, while abstract and full-text screening was independently commenced by EM and MA. Consensus was reached through discussion between the two reviewers at every level of the screening, where there was disagreement. During the title screening phase, articles which were not clear were passed on for abstract screening; if the abstracts were still not clear, they were included for full-text screening. These articles for full-text screening were screened by the two independent reviewers, as mentioned above, and guided by the inclusion and exclusion criteria. The reviewers resolved all discrepancies between them through consensus. The Kappa Statistic Framework was used to estimate the degree of agreement between the reviewers at full-text screening. Figure 1 shows the flow chart of the reviewed articles.

## Data extraction

A data extraction form was first designed by EM prior to the extraction of the data, to expedite the process. The main outcome of the review study is presented in Table 2. In order to answer the research question, the following information was extracted from the included articles: Name of the author(s) and date of publication; location of the study; study design; study setting; aim of the study; study population; sample size; findings of the study and the recommendations from the study.

**Table 2.** Characteristics, context, aim, study design and quality appraisal of included studies.

Author and date	Location of the study	Aim/s of the study	Study design	Sample size	Quality appraisal score (%)
Adam, 2014	Kenya	To study the effectiveness of a volunteer community health worker project that utilised a health prevention and promotion role for CHWs.	Quasi-experimental design	83	100
Agrawal, 2011	India	(i) To establish the relationship between the knowledge level of CHWs and auxiliary nurse midwives (ANMs) and antenatal home visits coverage, and (ii) to find out the effect of their knowledge level on essential newborn health care practices at the household level.	Mixed method	388	100
Austin-Evelyn, 2017	South Africa	To provide a preventive and health-promoting community-based PHC model.	Mixed method	91	
Brown, 2006	Peru	To describe the profile of community health workers in rural Quechua communities from Ayacucho, Peru.	Mixed method	171	
Cordeiro, 2015	Brazil	To describe and analyse the work of CHWs while focusing on the development of primary health care practices related to harmful drug consumption.	Qualitative	18	100
de Moura Pontes, 2011	Angola	To know the perceptions and practices of the Angolan community health agents (CHAs) regarding their work and the health of the population.	Mixed method	640	
Gauteng DoH, 2016	South Africa	(i) To understand the needs of the community; (ii) To advocate for the people in the community.	Not applicable	Not applicable	
Doherty, 2016	South Africa	To describe the high-impact interventions for pneumonia and diarrhoea which are now leading causes of under-5 mortality.	Quantitative	126	50
Florindo, 2014	Brazil	To describe the methodology for training to provide counselling on physical activity among CHWs working within primary health care in Brazil.	Quantitative Experimental design	65	100
Javanparast et al, 2011	Iran	To explore the perceptions of CHWs regarding their contribution to rural health in Iran.	Qualitative	91	100
Koyio et al, 2014	Kenya	To assess the knowledge and opinions of CHWs regarding HROLs and other oral diseases.	Mixed method	862	
Le Roux et al, 2015	Eastern Cape, South Africa	To describe how we have addressed the challenges of *Zithulele* in the Eastern Cape of South Africa over the last three years.	Quantitative	50	75
Lightspeed, 2015	South Africa	To determine the issue of proper employment and integration of CHWs in the Free State.	Not applicable	Not applicable	
Lindblade et al, 2006	Kenya	To evaluate the diagnostic accuracy of the WHO Haemoglobin Colour Scale (HCS) for anaemia in three groups of children aged 2 months to 2 years.	Quantitative study	793	100
Mukherjee and Eustache, 2007	Haiti	To explore the roles of the CHW within the programme of HIV prevention, antiretroviral treatment and the utilisation of AIDS and PHC services.	Mixed method	163	80
Negotiated Service Delivery Agreement, 2012	South Africa	(i) To strengthen the district health system (DHS) and (ii) to do the basics better.	Not applicable	Not applicable	
Nxumalo et al, 2013	Eastern Cape and Gauteng, South Africa	(i) To focus on the challenges faced by the South African government in addressing increasing health inequities and (ii) to ascertain the current efforts to strengthen primary health care (PHC) through CHWs’ outreach teams.	Qualitative	15	100
Perez et al, 2009	Mali	To assess the performance of CHWs in the promotion of basic child health services in rural Mali.	Mixed method	473	100
Perry, 2013	Ethiopia, Malawi, Pakistan and Brazil	To describe the achievements of ‘Health for All’ by the year 2000 through PHC.	Not applicable	Not applicable	
Prinja et al, 2014	India	To bridge the cost of delivery of a platform of health care services delivered through the CHWs at sub-centre (SC) level.	Quantitative	50	
Rennert and Koop, 2009	Honduras	To develop a model for the development of sustainable PHC in village communities in Honduras through the training and support of CHWs.	Quantitative	70	75
Roberton et al, 2015	Tanzania	(i) To explore the experiences of CHWs, supervisors, and village leaders involved in the Integrated MNCH Programme and (ii) to understand the initial strengths and challenges of its CHW supervision model and (iii) to offer further insight into innovations that support CHWs.	Mixed method	228	100
Signorelli et al, 2018	Brazil	To describe through ethnographic qualitative research within one specific BHU the intersections between federal policies, the SUS, and professionals who care for women living with domestic violence (DV) in PHC settings.	Qualitative	15	100
Sodo and Bosman, 2017	Limpopo Province, South Africa	(i) To determine the progress of WBPHCOTs, and (ii) to determine which aspects of the programme need to be improved.	Quantitative methods.	151	75
Sommanustweechai et al, 2016	Myanmar	To assess the socio-economic profiles’ contributions of CHWs to primary health care services and their need for support to maintain their quality contributions in rural hard-to-reach areas in Myanmar.	Quantitative study	715	100
Suri et al, 2007	KwaZulu-Natal, South Africa	To identify ways of improving the current CHW programme to more effectively combat the spread of HIV infection and TB.	Quantitative study	120	100
Tilahun et al, 2017	Ethiopia	To examine training needs and perspectives of community health extension workers (HEWs) in relation to providing child mental health care in rural Ethiopia.	Quantitative study	104	100
Van Ginneken et al, 2010	South Africa	(i) To explore the factors affecting the late apartheid projects’ evolutions, (ii) to note the extent to which this historical analysis intends to contribute to current debates on the appropriateness, effectiveness and sustainability of CHW initiatives within South Africa and to similar global debates.	Qualitative method	21	100
Whyte, 2015	Johannesburg, South Africa	To evaluate the implementation of WBOTs against national guidelines and identify (CHWs’ characteristics that influence adherence to guidelines regarding the referral and follow-up of maternal and child health clients.	Quantitative study	87	
Wilford et al, 2018	KwaZulu-Natal, South Africa	To explore the quality of CHW household visits to mothers and children using observations and in-depth interviews with mothers and CHWs. (As far as we are aware, this is the first study in South Africa to directly observe and assess care provided by CHWs in households).	A qualitative study design	45	100
Zulu, 2016	KwaZulu-Natal, South Africa	To evaluate the management and performance systems in order to provide effective and efficient PHC of rural ward-based primary health care in the UThukela District Municipality (UDM).	A mixed methods approach	385	

## Quality appraisal of the included articles

The Mixed Method Appraisal Tool (MMAT), 2018 version [15], was adopted to assess the quality of the included articles. The MMAT allowed the use of quantitative, qualitative and mixed methods study designs when evaluating the articles on primary research. This guide enabled the reviewer to assess the quality of the methods used in the included articles and to score each article between 25% and 100%, for each to be rated as being of high or low quality. A score between 25% and 49% was rated as being of low quality; a score between 50% and 74% was rated as being of average quality; and between 75% and 100% as high quality.

## Collation and summary of the findings

The data extraction was analysed using thematic analysis, based on the following pre-determined themes: The roles and responsibilities of community health workers who worked in PHC or were WBPHCOTs, as perceived by CHWs; the factors influencing the effectiveness of WBPHCOTs and the factors that negatively influenced the effectiveness of CHWs in WBPHCOTs. A thematic analysis was conducted, which included the following three of six steps: familiarisation with the data, generation of codes and the production of a report, as described by Braun and Clarke [16]. This helped the authors to make a thorough reflective and deliberate description of the themes in this study [16,17]. The authors jointly evaluated the themes and conducted a critical appraisal of each theme in relation to the research question. In addition, the meaning of the findings was scrutinised in relation to the aim of the study and the implications for policy and practice.

### Results

Out of the 69,969 articles retrieved from 5 electronic databases and 9 articles from other sources, only 31 (27 primary research and 4 grey literature) met the inclusion criteria for the study (Figure 1). The degree of agreement between screeners at the full-text screening stage was estimated and an inter rater reliability analysis was also performed, using the Kappa’s statistic with SPSS version 25 to determine the consistency between the reviewers. The level of agreement between the reviewers was 85.2%, which showed a fair level of agreement (Kappa statistic = 0.40, p-value<0.001). McNemar’s chi-square statistic suggested that there was not a statistically significant difference in the proportions of yes/no answers by the reviewers.

### Characteristics of included studies

Table 2 reveals that out of the 31 articles included in the study, 13 studies were conducted in different provinces in South Africa.

Of the South African studies, three were conducted in KwaZulu-Natal province [18–20], one was conducted in the Eastern Cape and one in Gauteng [21], two each in the Eastern Cape Province [22,23] and Limpopo Province [24], one in the Western Cape Province [25] and one in Johannesburg, the largest city in Gauteng Province [26]. Four of the South African studies did not specify the province [27–30]. However, the focus of this study was not to compare different provinces in South Africa.

The remaining 18 articles reported studies conducted in other parts of the world: Three studies were conducted in Kenya [31–33]; and two each in India [34,35] and Brazil [36,37] respectively.

In addition, one study each was conducted in Peru, a country in South America [38], Angola [39], Iran [40], Haiti [41], Mali [42], Honduras, a country in Central America [43], Tanzania [44], Myanmar in Asia [45] and Ethiopia [46].

Of the included articles, seven studies employed a qualitative study design [19,21,30,37,39,40,47], seven were quantitative randomised controlled (trials) studies [18,22,26,33,35,45,46], five were quantitative nonrandomised studies [24,25,31,36,43] and nine were mixed methods studies [20,32,34,38,39,41,42,44,48]. The methodological appraisal of the quality of the studies included rated each one as being of ‘high’ quality, as the scores for each were between 75–100% (Table 1).

### Factors positively influencing the effectiveness of WBPHCOTs

Table 3 presents the factors positively influencing the effectiveness of CHWs in WBPHCOTs and also describes the CHWs’ roles and responsibilities. Twenty-six of the reviewed studies identified diverse training as an essential factor for the CHWs’ to achieve effectiveness in WBPHCOTs [18,19,21,25–34,36-43,45–49]. The other enabling factors were the following: building of lasting and sustainable relationships with communities based on trust, recognition and encouragement [40,41]; building relationships with health care workers at different levels of the health care system; engaging and establishing a good relationship with families; having shared goals and supportive clinics; and having good, supportive hospital leadership [22]; clearly defined roles for CHWs [40]; and a mentoring and supervision programme [21,29] which should assist CHWs, particularly with their problem-solving and reporting skills [21]. One article reported that means of transport, adequate supervision and motivation of CHWs through the introduction of financial incentives and remuneration were among the key factors in improving the work of CHWs in rural communities [42]. Other studies found that support from facility-based supervisors and the visits of their supervisor to their village [18,19,26,44]; good leadership and supervision [30]; high motivation and the willingness of community health extension workers (HEWs) to apply and maintain determination in their tasks; having a positive attitude [46]; improving openness to partnerships; co-operation between the various partners and sectors and the provision of consumables [24]; as well as regular feedback to the respondents and consistently conducting performance reviews [20] were reported to be enabling factors.

**Table 3.** Enablers and barriers of community health workers’ effectiveness.

Author and date	Factors positively influencing effectiveness of CHWs in WBPHCOTs	Factors negatively influencing effectiveness of CHWs in WBPHCOTs	Results	Significance of the study	Conclusion/Recommendation
Adam, 2014	Training in skills practice.	Inadequate manpower (CHWS).	In each of the three separate areas where CHWs were trained, the number of women delivering with skilled attendance by CHWs was higher among those mothers who reported receiving at least one health message, compared to those who did not.	Knowledge of CHWs may promote delivery with skilled attendance by CHWs, which is essential.	The finding supported the Kenyan policy to promote health through a direct person-to-person trust-based spread of health messages. The ratio was 1 CHW to 20 patients, as recommended by the government.
Agrawal et al, 2011	Improving the knowledge level of CHWs through regular education and field-based refresher training programmes.	Poor knowledge.	The better the knowledge of CHWs or ANMs, the greater the number of women visited by them.	Better knowledge levels led to a greater number of patients seen for antenatal visits, and the women visited by this group showed adherence to essential newborn care practices at the household level.	Knowledge level of CHWs was an important factor.
Austin-Evelyn et al, 2017	Regular training programme, field-based supervision from the nurse team leader and availability of working tools in the field.	Overworked, insufficient supervision and CHWs struggled with their roles and scope of work.	Revealed the knowledge and perceptions of CHWs in PHC.	Misconceptions about CHWs’ roles and responsibilities by some community members.	Improving community health and well-being through programme management, supervision, scope and quality that challenged their ability to deliver on the potential of CHWs.
Brown et al, 2006	Training of community health workers.	Knowledge about health care needs and service provision in rural settings was lacking, with limited access to health care and geographical isolation of these indigenous people.	CHWs, although having limited education, were the most visible health care providers in the community.	Community health workers, with higher educational levels but also with higher drop-out rates.	The training of CHWs needed to incorporate culturally appropriate elements, as well as employ specific and simple educational techniques, to improve linkages between community health workers and health professionals.
Cordeiro and Soares, 2015	Training in health and technical health.	Difficulties related to the hierarchical structure and lack of credit from the technical team, among the other issues concerning the work process. Complex factor of work fragmentation in the process of the provision of health care diminished the role of workers. Low remuneration.	CHWs practiced a repetition of content taught to them in a hurried manner, drawn from clinical knowledge of the training of the nurses and physicians in universities.	Social abandonment of CHWs area and proposed complex practices. The role of the CHWs was not clearly defined.	High mortality rate of children due to lack of access and inadequate health services and shortage of manpower. To stimulate and facilitate access to health services.
de Moura Pontes et al, 2011	Follow-up visit.	There was no training of supervisors. Inadequate manpower.	CHAs in Angola perceived themselves as a ‘link’ between the community and health services.	CHWs should have a good relationship with the community and be acknowledged by the district coordinators and residents in the community.	Act on behalf of others. To implement health services.
Gauteng DoH, 2016	WBOT training for CHWs, in- service training.	Household members refused help. Clients gave wrong addresses, to health services who then could not trace them.	To identify the health needs of the people, especially the vulnerable.	Ward-based outreach team and social development.	Reduction in child mortality could be dramatically improved if there were more CHWs who were allowed to provide more health care.
Doherty et al, 2016	A higher CHW-to-population ratio than elsewhere. Expansion of the role of CHWs. An equity-focused strategy to train, supply and supervise CHWs to diagnose and treat diarrhoea, malaria and pneumonia in communities where access to health services was poor.	Low CHWs-to-population ratio, poor access to care.	Evidence showed that CHWs in sufficient numbers could have a rapid and positive impact on reducing neonatal and young child mortality, especially when they were allowed to treat common acute conditions.	The proposed role for CHWs in SA was extremely narrow, focusing primarily on counselling around prevention activities and adherence support. The role and scope of CHWs should be extended.	The creation of a period during work time dedicated to self-care and the practice of physical activities for the community health workers.
Florindo et al, 2014	Adequate information and knowledge on health issues.	Inadequate knowledge due to limited training given to CHWs.	The health workers developed a broader perspective of promoting physical activity in the context of health promotion and improvements in professionals’ perceptions regarding the benefits of physical activity for health. Better performance in terms of knowledge of physical activities for special cases, such as patients with cardiovascular, metabolic and bone diseases.	Although the health workers felt promoting physical activity was important, their approach was restricted to diseases and difficulties implementing actions that involved physical activity.	Community health workers needed to be integrated into the mainstream health care delivery system.
Javanparast et al, 2011	Building of lasting and sustainable relationships with communities, based on trust and recognition. Sound health knowledge and skills were the most important factors facilitating successful implementation of the CHW programme in Iran. The role of the CHW was to be clearly identified.	The heavy workload, lack of a support system, and poor supervisory mechanisms were the most common barriers.	CHWs were responsible for a wide range of activities because they had an in-depth understanding of health.	Training, programme enhancement and the forging of relationships with community members could be applicable to programmes in other countries seeking to improve the retention and performance of CHWs.	Supervision-related mechanisms (e.g., how supervisors could support CHWs to improve their performance).
Koyio et al, 2014	Training for building competence.	Lacked the appropriate knowledge required.	This probably indicated that the CHWs lacked the competence and skills needed for educating the community and mobilising it to seek oral health care services.	Development of a training course for increasing their knowledge.	CHWs needed to be educated about general oral and HIV-related oral diseases, early identification of (HIV-related) oral lesions and referral of community members suspected of being HIV-positive to the HFs.
Le Roux et al, 2015	Building relationships with health teams at different levels of the health care system, as well as having shared goals and supportive clinic and hospital leadership. Training on how to engage and establish a good relationship with a family.	Lack of a support system.	Commitment and excellence in health delivery by integrating care and training within the district.	Attitudes of families.	Integrating CHWs with PHC clinics and hospital health teams to improve maternal and child health that has had success in its early stages in a rural area.
Lightspeed, 2015	The imparting of basic knowledge to community health workers in the programmes (**WBPHCOTs)**.	Population density; burden of disease in catchment population, and the distance from a primary health care facility.	The employment of CHWs.	It was important to involve lecturers in the implementation plan (IP) because most of the students (qualified) became the team leaders of the outreach teams.	To integrate CHWs into ward-based primary health care teams.
Lindblade et al, 2006	Training programme.	Inadequate knowledge and skill.	The prevalence of anaemia was not significantly different between the two groups of children recruited from the health facilities (P ¼ 0.25), but was significantly different between the children attending the health facility and children from the general population (sick children).	In areas of high anaemia prevalence, the HCS could increase the recognition and treatment.	According to the WHO, HCS was neither the best method for diagnosing anaemia, nor the least expensive, but could be the most economical method.
Mukherjee and Eustache, 2007	Trust, encouragement, better understanding of the disease.	Feelings of dissatisfaction with their salary.	The CHWs perceived that they had an important role in increasing access to care, particularly among vulnerable groups. They perceived their role as a strong promoter of the integration of the medical aspects of the disease with the spiritual components, particularly in providing emotional support and helping affected persons discuss and disclose their status to their families.	Understanding the importance of the CHWs in encouraging service utilisation. Nearly all of the patients attending the clinics were rural subsistence farmers. The average length of time a family walked to the Lascahobas clinic was three hours. Since the CHWs are themselves from the community, they often accompanied patients, families or even groups of patients from villages to the clinic.	The important factors of the CHWs’ work were psychosocial support and community solidarity; which should be given greater focus during training and supervision.
Negotiated Service Delivery Agreement, 2012	Skills competence development through an extensive orientation, training, mentorship and supervision programme. A team approach which included community health workers (CHWs).	Inadequate mentorship from professionals and from their training.	CHWs would also provide psycho-social support and manage interventions such as treatment, defaulter tracing and adherence support.	Implementation of policy and CHWs to facilitate access to health and other services.	Community assessments, community and group interventions.
Nxumalo et al, 2013	Training, supervision, and mentoring to assist CHWs, particularly in problem-solving skills and reporting.	The fragmentation and resultant lack of coordination within and between government departments at all levels was a common and significant constraint to improving access in all three communities. The lack of political accountability across all case studies had a detrimental effect on CHWs’ service.	Understanding social determinants as a cause of poor health was key to shaping the role and services of CHWs. This was conceptualised within the health sector and CHW activities that were confined to health issues.	The clients of CHWs often did not have identity documents and birth certificates which were required to obtain their social benefits.	The success and sustainability of CHW programmes required the ongoing commitment of resources, including investment in quality training, supervision, mentoring, and organisational support. In addition, resources were needed. The national programme of PHC outreach teams in South Africa was unlikely to achieve its expected outcomes unless there was sufficient capacity to support CHWs to operate effectively at the interface between the community and the health system.
Perez et al, 2009	Continuous training, having access to transport, adequate supervision and motivation of CHWs through the introduction of financial incentives and remuneration were among key factors to improving the work of CHWs in rural communities.	Inadequate resources, low coverage of the CHWs.	The study evaluated knowledge and practice concerning home management of fever and diarrhoea among infants and children under the age of 5 as a proxy indicator of the performance of CHWs at the household level. Results indicated that correct management of fever had been relatively good (40%). In contrast, management of diarrhoea was poor.	When compared to knowledge and practice, a positive influence of CHWs on specific essential family health practices by the households was found, namely knowledge of the management of childhood fever.	Reinforcing the role of CHWs could facilitate the improvement of child health when strategies such as upgrading existing lower-level facilities, improving and building referral systems, training and supervision were considered.
Perry, 2013	Training in integrated community case management (ICCM) and the diagnosis and treatment at the community level of childhood pneumonia, diarrhoea and malaria.	Role conflict between other health workers. Expansion in the LHWs’ roles and tasks had increased their workload.	Transformative agenda.	The effective functioning of large-scale CHW programmes offered one of the most important opportunities for improving the health of impoverished populations in low-income countries.	Decision-makers and programme implementers considered the initiation, expansion or strengthening of CHW programmes in their country.
Prinja et al, 2014	Human resource costs.	Delivery load excessive, inadequate manpower.	To provide health services through CHWs at sub-centre level. Cost of human resource alone accounted for 58%, followed by drugs (18%) and capital (13%). Almost half of the cost was incurred in the provision of services as part of an outreach programme, while 40% of the resources were spent on delivering services in an out-patient setting.	The tolerance value and VIF ranged between 0.535–0.845 and 1.18–1.86 respectively, indicating an absence of multicollinearity. Controlling for other determinants, we found that a 10% increase in human resource cost led to a 6% (p, 0.001) increase in the cost per person per year.	Our estimates would be useful in undertaking full economic evaluations or equity analysis of CHW programmes.
Rennert and Koop, 2009	Training, monitoring CHW performance, maintaining a high level of health worker training and providing continuous support to the CHWs.	Inappropriate case management was noted.	Follow-up visits by brigade evaluation teams documented a significant improvement in CHW patient assessment and prescribing behaviour over time.	The potential differences in the performance and community acceptance of male versus female CHWs, as well as problems and challenges around CHW reimbursement.	Ongoing evaluation, supervision, in-service training, and guidance were essential to maintain a successful health worker programme.
Roberton et al, 2015	The visits of their supervisor to their village. Support from facility-based supervisors.	Limiting the opportunities for one-on-one mentoring and individual feedback. Facility-based supervisors did not visit CHWs in their villages often, and supervision visits from district and regional staff were infrequent and scheduled with little advance notice.	The findings suggested that CHW supervision focused primarily on accountability and report checking. CHWs overwhelmingly said they felt positive about supervision and appreciated the support offered by facility-based supervisors. The supervisors themselves also spoke positively about supervision as an opportunity to provide feedback and support to CHWs.	Unrealistic expectations of what facility health workers were able to achieve, given human resource shortages and social constraints.	Supervision of CHWs could be strengthened by streamlining supervision protocols to focus less on report checking and more on problem-solving and skills development.
Signorelli et al, 2018	Appropriate training.	Inadequate training.	Public policies and their implementation in locally relevant PHC services and the potential key role of CHW in providing care for women experiencing domestic violence (DV). CHWs constantly visited people under their care, entering the domestic space so that dialogue could be established spontaneously and horizontally, though this was not always easily achieved.	Gaps in training/awareness with a lack of effective strategies for combating unequal gender relations.	This Brazilian experience could constitute a key strategy to support women affected by DV, both in chronic and acute situations. Listening to professionals and para-professionals, who were in direct contact with women victims of DV was essential to illuminate theory, policies and practices.
Sodo and Bosman, 2017	Openness to partnerships, co-operation between the various partners and sectors, and provision of consumables.	Resources such as stationery, equipment batteries and transport to conduct household visits. Poor planning and the lack of a budget for WBOTs. It affected the proper implementation of the programme and could result in poor outcomes.	The results of the assessment reported that 71 413 household visits were conducted in the financial year 2014/2015. The evidence showed that the programme contributed to strengthening linkages to other sectors and departments through a referral system. The results of the assessment reported that the professional nurses who worked full time in the facilities were delegated to perform the OTL’s duties, but they did not have enough time to go out and support the teams due to gross staff shortages in the facilities.	The use of delegated human resources was unrealistic because it affected the supervision of the programme.	The programme was achieving its set target, although there were still some problems in implementation, such as the dual roles played by the outreach team leaders and CHWs.
Sommanustweechai et al, 2016	Retraining, supervision and support would prevent them from becoming ‘quacks’ while maximising their potential contributions.	Inadequate support, in particular technical supervision, as well as the replenishment of CHW kits and financial support for their work and transportation.	CHWs were able to provide some of the services by themselves, such as the treatment of simple illnesses and they had provided services to 62 patients in the preceding 6 months. Their contributions to primary health care services were well accepted by the communities as they were geographically and culturally accessible.	The CHWs’ confidence in providing health services was positively associated with their age, education, and more recent training.	Given their contributions and easy access, policies to strengthen support to sustain their contributions and ensure the quality of services were recommended.
Suri et al, 2007	Training and support for CHWs.	Perceived lack of confidentiality; and inability to pay for transportation to the clinic.	Results suggested that CHWs recognised the need for HIV/AIDS- and TB-related interventions, but were unable to provide a response commensurate with this need. CHWs ranked HIV/AIDS as the highest priority among 8 pre-identified concerns facing the community.	Contradictory sentiments regarding the objectives and capability of the current CHW programme were expressed by the academic community.	In order to fully enable the existing CHWs’ programme to effectively fight the HIV/AIDS and TB co-epidemics, substantial improvements in supervision and collaboration had to be made in KwaZulu-Natal.
Tilahun et al, 2017	High motivation and willingness of HEWs to apply and maintain effort in their tasks; positive attitude. Improving competence and knowledge through training.	Inadequate training, poor knowledge and skills, negative attitudes, demotivation and institutional constraints.	Limited training in the mental health needs of children. The number of mental disorders among children in the community was considered high.	Opportunities mentioned included staff commitment, high levels of interest and a positive attitude toward providing the service.	If the key barriers to service provision were addressed and supported by policy guidance, community health workers could contribute substantially by addressing the treatment gap for children with mental health needs.
Van Ginneken et al, 2010	Good leadership and supervision, even though not always achieved, were essential to the success of programme.	High expectations of health services by the community.	The strong socio-political motivations of the late apartheid period projects were mostly not carried through into the post-apartheid period. The current struggle to redress the economic, health and racial inequalities had not been effective.	Poorly addressed issues, particularly in larger scale initiatives.	CHWs programmes within South Africa and globally, lessons learned from past programmes should play a stronger role in informing current policies.
Whyte, 2015	In-service training and support.	Lack of supervision, limited resources and poor knowledge and resources required to conduct household visits.	CHWs adhered to the guidelines regarding the follow-up of maternal clients with 85% of CHWs having conducted the required number of follow-up visits for pregnant and postnatal women; only 29% of children received follow-ups.	Both CHWs and supervisors needed ongoing training and supervision.	More resources should be available, CHWs’ supervision, capacity and training to improve the implementation process of future teams.
Wilford et al, 2018	Training and support from supervisors; provide services for mothers and children in the household.	Inadequate training and supervision of the gaps in CHWs’ knowledge and skills.	There were important gaps in the content provided by CHWs. Mothers expressed satisfaction with CHWs’ visits and appreciation that CHWs understood their life experiences and therefore provided advice and support that was relevant and accessible. CHWs expressed concern that they did not have the knowledge required to undertake all activities in the household and requested training and support from supervisors during household visits.	Training should include practical skills components, rather than only the classroom-based instruction currently available in South Africa. Although CHWs were well-accepted and appreciated by the mothers they visited, the care they provided was sub-optimal with many missed opportunities to provide important health information and to identify important health issues in the household.	A comprehensive and sustainable package of skills development, support and supervision of CHWs is required if this cadre is to reach their full potential and provide effective care for mothers and babies in the community.
Zulu, 2016	Regular feedback to the respondents and conducting performance reviews consistently.	Lack of management and supervisory support contributed to high rates of dissatisfaction amongst CCGs, as well as poor quality of work for community caregivers.	Ward-based outreach teams were crucial in the delivery of PHC services in rural municipal wards within the Operation *Sukuma Sakhe* programme. The lack of management and supervisory support contributed to high rates of dissatisfaction amongst CCGs, as well as poor quality of work by the community caregivers. There was a need for the Department of Health (DoH) to invest in the ward-based outreach teams (WBOTs) and allocate CCG budgets within the ward-based outreach teams.	The formulation of policies, programmes, methods and interventions which would enable the UThukela District Municipality to improve health outcomes.	The monitoring and evaluation policy should be reviewed to state clearly the tools, activities and benefits of the implementation of the M & E performance management systems. The use of point-of-care technology by the WBOTs should be strengthened, especially in deep rural wards.

According to Javanparast et al [40], sound knowledge and skills were the most important factors facilitating successful implementation of the CHWs’ programme in Iran. Places where CHWs were trained on skills practice were reported to have a higher number of women delivering less-skilled performance [31]. CHWs’ regular education and field-based refresher training programmes [29,34,42,45] resulted in increased knowledge levels [34] and skills competence development [29], which resulted in better patient coverage during antenatal visits [34]. To improve the CHWs’ effectiveness, their training needed to incorporate culturally appropriate elements and employ specific and simple educational techniques to improve the linkages between community health workers and health professionals [38]. There was a need to train CHWs in health and technical health as they practiced a repetition of content taught in a hurried manner [47]. CHWs needed to have adequate information and health-related knowledge [36,41] which would enable them to build on their competencies [32,46]. Child mortality could be dramatically reduced if there was in-service training for CHWs and if they were allowed to do more when assessing patients [27]. A higher CHW-to-population ratio, the expansion of the role of CHWs and an equity-focused strategy to train, supply and supervise community health workers (CHWs) to diagnose and treat acute diseases in communities where access to health services was poor [25] would further increase child survival rates. Hence, it was deemed essential to impart knowledge to the CHWs and integrate them into ward-based primary health care teams [28]. Training of CHWs on integrated community case management (ICCM) was a programme which ensured effective functioning of a great number of CHWs to improve the health of impoverished populations in low-income countries [49]. Retraining, supervision and support prevented them from becoming ‘quacks’ while it maximised their potential contributions [45]. Improved knowledge of CHWs could promote the prospect of them delivering of babies under skilled supervision [31]. Additionally, women visited by CHWs who received regular education and attended field-based refresher training programmes, showed adherence to essential newborn care practices at the household level [34].

### Factors that negatively influenced the effectiveness of CHWs in WBPHCOTs

There were many factors reported in the various studies identified that diminished the effectiveness of CHWs in WBPHCOTs. Some of these were inadequate manpower (CHWS) [31], poor knowledge [34] due to limited training given to CHWs [36], and being overworked and not being adequately supervised [48]. A lack of knowledge about health care needs and service provision in rural settings, as well as limited access to health care and the geographical isolation of indigenous people also reduced the effectiveness of CHWs in WBPHCOTs, according to one study [38]. Experiencing difficulties related to a hierarchical work structure and a lack of credit from their technical team were found to be some of the issues affecting CHWs’ effectiveness, as was the complexity of some work processes, the fragmentation of duties in the health care process as a whole, and low remuneration rates [47]. Inadequate training of supervisors, inadequate manpower and lack of training of CHWs was reported [39], as was a low CHWs-to-population ratio, poor access to care, and poor remuneration once again [25,41]. Another study identified a heavy workload, the lack of a support system and poor supervisory mechanisms as impacting on the services provided by CHWs [40]. Other studies identified a lack of appropriate knowledge and support [32,33], inadequate mentorship from professionals, insufficient training, fragmentation and the resultant lack of coordination within and between government departments at all levels, and a lack of political accountability as playing a role [21,29]. Some authors reported inadequate resources, low coverage of the CHWs [42], and role conflict due to other health workers’ expansion into the CHWs’ roles and tasks as having increased the workload of CHWs and diminished their effectiveness [49]. Again, a heavy workload, insufficient manpower, insufficient resources such as stationery, equipment, batteries and transport to conduct household visits, poor planning and the lack of a budget for WBOTs had a detrimental effect on CHWs’ services.

Facility-based supervisors did not often visit CHWs in their villages, and supervision visits from district and regional staffs were infrequent and scheduled with little advance notice [35,44]. Inadequate support exemplified by poor technical supervision, CHW kits not being replenished, lacking financial support for their work and transportation [45], and a lack of management and supervisory support contributed to the high rates of dissatisfaction amongst community caregivers (CCGs), as well as poor quality of work by the effected community caregivers [20]. A perceived lack of confidentiality and the inability to pay for transportation to the clinic [18], inadequate training, poor knowledge and skills, negative attitudes, demotivation, institutional constraints [46] and high expectations of health services were also identified as impacting on the service delivery of the CHWs [30].

The lack of supervision, limited resources and poor knowledge and resources required to conduct household visits [26]; inadequate training and supervision [19], lack of management and supervisory support and poor quality of work for community caregivers [20] were also factors affecting the CHWs’ impact.

All of the above factors affected the proper implementation of the programme and could result in poor outcomes [24]. Regardless of which country was examined, what was obvious was that the various studies cited above reported many challenges faced by CHWs, and many of these challenges were similar.

### Perceived roles and responsibilities of community health workers who were members of WBPHCOTs

As the role of the community health worker has not been clearly defined, this leads to the social abandonment of CHWs’ areas and proposed complex practices [47]. Doherty, Kroon and Rhoda et al [25] reported that CHWs had an extremely narrow role and scope in SA. Javanparast, Baum and Labonte et al [40] reported that CHWs were responsible for a wide range of activities because they had an in-depth understanding of health and the CHWs themselves perceived that they had an important role in increasing access to care, particularly among vulnerable groups. They perceived their role as being a strong promoter of the integration of the medical aspects of a disease with the spiritual components, particularly in providing emotional support and helping affected persons discuss and disclose their status to their families [41]. They saw themselves as providing direct person-to-person trust-based dissemination of health messages [31] when conducting home visits [19,34,37]. They also perceived themselves as mediators between the community and health services [39]. They provided primary health care services that were well received by the community [45], such as counselling around health promotion and disease prevention activities, and supported adherence to treatment [25,36]. They also provided psychosocial support [29,41]; promoted community solidarity [41]; and managed interventions such as treatment defaulter tracing and adherence support [29].

It was suggested that the role and scope of CHWs be extended [25] and integrated with PHC clinics and hospital health teams to improve maternal and child health, and this has had success in its early stages in trial in a rural area [22]. CHWs recognised the need for HIV/AIDS and TB-related interventions, but were unable to provide a response commensurate to this need [18]. If they had adequate training and knowledge, community health workers could also contribute substantially by addressing the treatment gap for children with mental health needs [46]. CHWs expressed concern that they did not have the knowledge required to undertake all activities in the household and requested training and support from supervisors when conducting household visits [19]. A comprehensive and sustainable package of skills development, support and supervision of CHWs was required if this cadre was to reach its full potential and provide effective care in the community [19]. Ward-based outreach teams were deemed crucial in the delivery of PHC services in rural municipal wards. The Department of Health (DoH) was to invest in the ward-based outreach teams (WBOTs) and allocate community caregiver (CCG) budgets within the ward-based outreach teams [20]. The national programme of PHC outreach teams in South Africa was unlikely to achieve its expected outcomes unless there was sufficient capacity to support the CHWs to operate effectively at the interface between the community and the health system [21]. Reinforcing the role of CHWs could facilitate the improvement of child health when strategies such as upgrading existing lower-level facilities, improving and building referral systems, training and supervision were considered [42].

## Discussion

This review has identified the factors that positively influence the effectiveness of community health workers (CHWs) as follows: Training which includes skills training and improving the CHWs’ knowledge through regular refresher training programmes, field-based supervision, follow-up visits and mentoring. Building of lasting and sustainable relationships with communities and health teams has also been identified as an enabling factor. These relationships should be based on trust and recognition, as well as on encouragement and capacitating the CHWs to understand diseases better. Extrinsic motivators such as financial incentives and remuneration are key factors to improving the CHWs’ work in rural communities. Other motivating factors include expansion of the role of the CHWs by training and capacitating them to diagnose and treat diarrhoea, malaria and pneumonia in communities with poor access to health services. Conversely, factors that negatively influence the effectiveness of CHWs are inadequate manpower, poor knowledge levels, being overworked, having insufficient supervision and struggling with their roles and scope of practice. Difficulties related to hierarchical structure and lack of credit from technical teams are also amongst the barriers to their effectiveness. Coupled with these are the complexities of work fragmentation and low remuneration. Household members refusing help and clients giving incorrect addresses also restrict the effectiveness of the CHWs in the health system, as these clients are usually lost to follow-up. Long distances travelled from the primary health care facility, population density and the high burden of diseases are additionally amongst the contributory factors that render CHWs ineffective. This is exacerbated by inadequate resources and low coverage of CHWs.

This review has also explored the perceived roles and responsibilities of community health workers and ward-based primary health care outreach teams working in a re-engineered primary health care system. Community health workers complement the overstretched health work force and they are key to increasing access to basic health services in remote areas, thereby bridging the health equity gap [10]. Community health workers enjoy their work, find it meaningful [50], and see themselves as forming a link between communities and health services [41]. Community health workers in ward-based outreach teams serve as health promoters and they provide primary health care services that are well received by the community [45]. These services include counselling around health promotion, disease prevention activities and adherence support [25,36]. The scope of work of CHWs in WBPHCOTs includes:

- Heath promotion and illness prevention;
- Community assessment and mobilisation around community needs;
- Household assessment to identify their health needs;
- Psychosocial support to community members;
- Identification and management of minor health problems;
- Supporting screening and health promotion programmes in schools and early childhood development centres;
- Intersectoral collaboration and joint efforts in establishing community-based interventions, and
- Supporting the continuum of care through service coordination with other relevant service providers [50].

However, there is still a need to strengthen the implementation of the WBPHCOTs through training and supportive supervision. Community health workers in WBPHCOTs were concerned about their lack of knowledge to carry out all health activities in the households, and they expressed the need for training and support during their household visits by supervisors [19].

The review highlights a number of factors enabling the effectiveness of CHWs in WBPHCOTs, and these are summarised as follows:

The training of CHWs on the skills required, in-service education of the WBOTS and retaining and improving their knowledge levels through regular education and field-based refresher training [18,22,26,27,31–34,37,38,47]; doing follow-up visits with clients [39]; having field-based supervision from the nurse team leader; the availability of the tools required for working in the field [48] and receiving support from facility-based supervisors [44].

The expansion of the CHWs’ role as being part of an equity-focused strategy to train, supply and supervise CHWs to diagnose and treat diarrhoea, malaria and pneumonia in communities where access to health services is poor should be considered. This also includes capacitating CHWs for integrated community case management [25,49].

Building lasting and sustainable relationships based on trust and recognition with communities [40], while emphasising relationship-building with health teams [22], could be part of the role of the CHWs. Shared goals and supportive clinic and hospital leadership will also make CHWs more effective.

Other enablers of their effectiveness include skills competence development through extensive orientation, training, mentorship, supervision and a team approach [29], mentoring in problem solving and reporting skills [21] and partnerships with other sectors [24]. While good leadership and supervision are essential for the success of CHWs and WBPHCOTs programmes [30], regular feedback and conducting performance reviews [20] are also of great importance.

The review has also identified many factors hampering the effectiveness of CHWs in WBPHCOTs. These can be summarised as poor knowledge due to limited training, overwork, inadequate supervision, inadequate mentoring, limited access to health care and geographic isolation, low remuneration and scarce resources.

## Recommendations

Further studies are needed on the knowledge and accountability of the community health worker, for continuity and sustainability of the WBPHCOTs. Therefore, it is recommended that the CHWs be given adequate training and supportive supervision, as per their job description, for optimal performance. Training needs to be scaled up at the different levels, for example: at the entry level for CHWs when they get employed for the job; ongoing on-the-job training; refresher training in the form of workshops; and regular updates in preparation for the various health campaigns. Further research is needed to explore supervision models and mentoring for CHWs. We also recommend programmes to raise awareness in the communities on the roles and responsibilities of CHWs, to facilitate their acceptance by community members. Raising of awareness should be done on an ongoing basis so that CHWs may earn respect from the communities they serve. There is also a need to motivate this group of health workers with fair remuneration, which could increase their motivation and job satisfaction.

## Strengths and limitations of the study

The systematic method of data gathering using different electronic databases and the use of Google forms to manage the data have strengthened the reliability of the study. In addition, the effectiveness of CHWs in WBPHCOTs is a road map to improve the quality of health care, as shown in this study. During the thematic analysis all authors were engaged in the analytical process and in the discussion of the themes. This is sometimes referred to as an analyst triangulation. For sustainable health coverage to be achieved, the government should provide enough manpower in WBPHCOTs to strengthen the health care services. A limitation has been the small number of studies on WBPHCOTs and CHWs in low- and middle-income countries, especially in Africa. Further studies should be conducted to explore how the nurses perceive CHWs.

## Conclusion

This scoping review has highlighted the perceived roles and responsibilities of community health workers in ward-based primary health care outreach teams. The review has further provided factors enabling and hindering the effectiveness of ward-based primary health care outreach teams in rural primary health care settings. The results from this review will be useful in informing the rollout of the National Health Insurance, which is currently being piloted in South Africa. This review has also highlighted the impact that ward-based primary health care outreach teams have on the national health care system and on the health of the population.

## Paper context

Community health workers are an important category of health care workers in the structure of ward-based primary health care outreach teams in South Africa. Community health workers assume a number of roles while providing health care to communities. This scoping review sought to explore the perceptions of community health workers’ roles and responsibilities, and their roles in ward-based primary health care outreach teams. This review identified the factors enabling and hindering the ward-based primary health care outreach team’s effectiveness.
